# Characterization of the acoustic community of vocal fishes in the Azores

**DOI:** 10.7717/peerj.7772

**Published:** 2019-11-04

**Authors:** Rita Carriço, Mónica A. Silva, Gui M. Menezes, Paulo J. Fonseca, Maria Clara P. Amorim

**Affiliations:** 1 Okeanos-UAc R&D Center, University of the Azores, Horta, Portugal; MARE - Marine and Environmental Sciences Centre and IMAR - Institute of Marine Research, Horta, Açores, Portugal; 2 MARE - Marine and Environmental Sciences Centre, ISPA - Instituto Universitário, Lisboa, Portugal; 3 Biology Department, Woods Hole Oceanographic Institution, Woods Hole Oceanographic Institution, Barnstable County, MA, United States of America; 4 Departamento de Biologia Animal and cE3c - Centre for Ecology, Evolution and Environmental Changes, Faculdade de Ciências, Universidade de Lisboa, Lisboa, Portugal; 5 Departamento de Biologia Animal, Faculdade de Ciências, Universidade de Lisboa, Lisbon, Portugal

**Keywords:** Passive acoustic monitoring, Azores, Seamounts, Fish sounds, Acoustic communication

## Abstract

Sounds produced by teleost fishes are an important component of marine soundscapes, making passive acoustic monitoring (PAM) an effective way to map the presence of vocal fishes with a minimal impact on ecosystems. Based on a literature review, we list the known soniferous fish species occurring in Azorean waters and compile their sounds. We also describe new fish sounds recorded in Azores seamounts. From the literature, we identified 20 vocal fish species present in Azores. We analysed long-term acoustic recordings carried out since 2008 in Condor and Princesa Alice seamounts and describe 20 new putative fish sound sequences. Although we propose candidates as the source of some vocalizations, this study puts into evidence the myriad of fish sounds lacking species identification. In addition to identifying new sound sequences, we provide the first marine fish sound library for Azores. Our acoustic library will allow to monitor soniferous fish species for conservation and management purposes.

## Introduction

Acoustic communication in fishes is widespread and occurs in different environmental and behavioural contexts (Zelick, Mann & Popper, 1999). More than 800 fish species are known to produce sounds (Fish & Mowbray, 1970; Rountree et al., 2006). These sounds are species-specific and are associated with courtship, spawning, parental care, feeding, aggressive or territorial behaviours, and can be effective indicators of fish species richness and diversity in biological processes (Fine, Winn & Olla, 1977; Amorim et al., 2008; Lobel, Kaatz & Rice, 2010). The majority of fish sounds are low frequency (<3 kHz, mostly <1 kHz) and are made up of repetitive elements such as sound pulses (Amorim, 2006). Sounds made by fish species differ in fundamental frequency, dominant frequency, number of pulses and frequently in pulse period (Amorim et al., 2008; Colleye et al., 2011; Ladich, 2013). Fish sounds also differ from those made by other marine organisms such as cetaceans. Fish sounds frequently have a short duration (<5 s), are made of broad-band pulses, and often present multiple frequency harmonics (Fish & Mowbray, 1970). Sounds produced by cetaceans range from low frequencies (<1 kHz) up to 200 kHz, frequently have a long duration (>10 s), and often exhibit strong frequency modulation (Richardson et al., 1995). For example, Odontocetes produce a wide variety of whistles and broadband sounds (clicks) with main energies ranging from a few kHz (thus well detectable by humans) to ultrasonic frequencies of >100 kHz for echolocating prey and screening the environment. Baleen whales produce high intensity, lower frequency sounds (mostly <1 kHz, but can reach 24 kHz) lasting less than 1 s to over 30 s, spanning from simple growls to loud complex modulated songs (Mellinger et al., 2007; Au & Hastings, 2008). Fish sounds also differ from invertebrate sounds; the latter are typically broadband pulses with frequencies between 2 and 12 kHz (Au, 1998; Radford et al., 2008; Bittencourt et al., 2016), although some invertebrates may also produce much lower frequency sounds (e.g.,  Staaterman et al., 2011; Di Iorio et al., 2012). These differences allow discrimination of fish sounds in marine soundscape studies and offer a non-invasive way (through acoustic monitoring) to assess biodiversity of acoustic communities (Farina & James, 2016). Here, we consider the definition of acoustic community proposed by Farina & James (2016), i.e., a temporary aggregation of species that interact acoustically either in aquatic or terrestrial environments, producing sounds with internal or extra-body tools.

Owing in part to cost reduction and to technological improvements, Passive Acoustic Monitoring (PAM) of fish sounds has been increasingly used (e.g.,  Wall, Lembke & Mann, 2012; Tricas & Boyle, 2014; Rice et al., 2016). Besides having a negligible impact on biota, PAM supports long-term field studies of seasonal activities, and has been shown to be a useful tool in the conservation and management of vocal species (Mann & Lobel, 1995; Luczkovich, Mann & Rountree, 2008; Parmentier et al., 2018). In addition, monitoring the diversity of sounds made by marine organisms, including fish, can help assess ecosystem health (Bertucci et al., 2016). But PAM also presents limitations in its effectiveness. PAM will only record animal sounds when their sound pressure levels (or particle motion levels) are higher than the ambient noise levels, which means that taxa that produce low amplitude vocalizations (e.g., gobies; Lugli & Fine, 2003) will not be detected. Most importantly, there is a paucity of data on the sounds made by fish in their natural habitat and numerous fish sounds have not yet been identified (Tricas & Boyle, 2014; Ruppé et al., 2015; Mouy et al., 2018).

**Table 1:** Summary of marine fishes of Azores that have been reported to produce sound.

**Species**^a^	**Family**^a^	**Conservation status**^b^	**Commercial status**^c^	**Depth range (m)**^b^^,^^c^
*Balistes capriscus* (Grey triggerfish)^3^	Balistidae	VU	Commercial; gamefish; public aquariums	0–100 (0-55)
*Caranx crysos* (Blue runner)^3^	Carangidae	LC	Minor commercial; gamefish	0–100
*Elagatis bipinnulata* (Rainbow runner)^3^	Carangidae	LC	Highly commercial; gamefish	0–150 (2–10)
*Naucrates ductor* (Pilotfish)^9^	Carangidae	LC	Minor commercial, gamefish, public aquariums	0–300
*Seriola dumerili* (Greater amberjack)^3^	Carangidae	LC	Minor commercial; aquaculture; gamefish; public aquariums	1–360 (18-72)
*Trachinotus ovatus* (Pompano)^3^	Carangidae	LC	Minor commercial; aquaculture; gamefish	50–200
*Dactylopterus volitans* (Flying gurnard)^3^	Dactylopteridae	LC	Minor commercial; gamefish; aquarium	1–100
*Diodon hystrix* (Porcupine fish)^3^	Diodontidae	LC	Minor commercial; aquarium	2–50 (3-20)
*Gobius paganellus* (Rock goby)^5^	Gobiidae	LC	Minor commercial; aquarium	0–15
*Pomatoschistus pictus* (Painted goby)^6^	Gobiidae	LC	No interest	1–55 (1-50)
*Kyphosus sectatrix* (Bermuda sea chub)^3^	Kyphosidae	LC	Minor commercial; gamefish; public aquariums	1–30 (1-10)
*Mola mola* (Sunfish)^3^	Molidae	VU	Minor commercial	30–480 (30-70)
*Abudefduf luridus* (Canary damsel)^1^	Pomacentridae	LC	Minor commercial	0–25
*Pomatomus saltatrix* (Blue fish)^3^	Pomatomidae	VU	Highly commercial; aquaculture; gamefish; bait	0–200
*Scorpaena plumieri* (Spotted scorpionfish)^3^	Scorpaenidae	LC	Minor commercial; aquarium	1–60 (5-55)
*Epinephelus marginatus* (Dusky grouper)^4^	Serranidae	EN	Highly commercial; gamefish	8–300 (8-50)
*Canthigaster rostrata* (Sharpnose puffer)^3^	Tetraodontidae	LC	Aquarium	0–40
*Chelidonichthys cuculus* (Red gurnard)^2^	Triglidae	LC	Minor commercial	15–400 (30-250)
*Chelidonichthys lastoviza (* Streaked gurnard)^7^	Triglidae	LC	Commercial	10–150 (10-40)
*Zeus faber* (John dory)^8^	Zeidae	DD	Commercial; gamefish; aquarium	5–400 (50-150)

**Notes.**

a Marine fishes of the Azores-Annotated checklist and bibliography.

b IUCN Red List of Threatened Species (2017)

c http://www.fishbase.org/

Sound production source:

1 Santiago & Castro (1997)

2 Amorim (1996)

3 Fish & Mowbray (1970)

4 Bertucci et al. (2015)

5 Malavasi, Collatuzzo & Torricelli (2008) and Parmentier et al. (2013)

6 Amorim & Neves (2008)

7 Amorim & Hawkins (2000)

8 Onuki & Somiya (2004)

9 Fish (1954)

Conservation status from IUCN: LC, Least Concern; VU, Vulnerable; NT, Near Threatened; EN, Endangered; DD, Data deficient; Depth range with most frequent depths presented in brackets. Commercial status: indication of the degree of commercial interest referring to fisheries followed by other types of commercialization. All species are found frequently in Azorean waters.

Seamounts and nearshore bank areas of the Azores are important hotspots of marine biodiversity, harboring fish species of commercial and conservation interest (Pitcher et al., 2007), several of which are known to vocalize (e.g., dusky grouper *Epinephelus marginatus*, ocean sunfish *Mola mola*, tarpon *Megalops atlanticus*, (Fish & Mowbray, 1970; Bertucci et al., 2015). Despite its large fish biodiversity (about 460 marine fish species were recognized in the Azores, (Santos, Porteiro & Barreiros, 1997), only 20 species present in waters of this archipelago have been reported as soniferous (Table 1) suggesting that more remain to be identified. Because seamounts are subjected to increasing anthropogenic pressure, caused by overfishing and marine pollution (e.g., plastic and noise pollution), it is paramount to develop effective tools, such as PAM, to monitor ecosystems in support of conservation and management. With this objective in mind we (1) listed the known vocal fish species occurring in Azorean waters and compiled their sounds; (2) characterized new sound types and proposed their association to known fish sounds; and (3) built a fish sound database to be used as a steppingstone both for future research and for conservation and management.

## Materials & Methods

### Study sites

**Figure 1:**
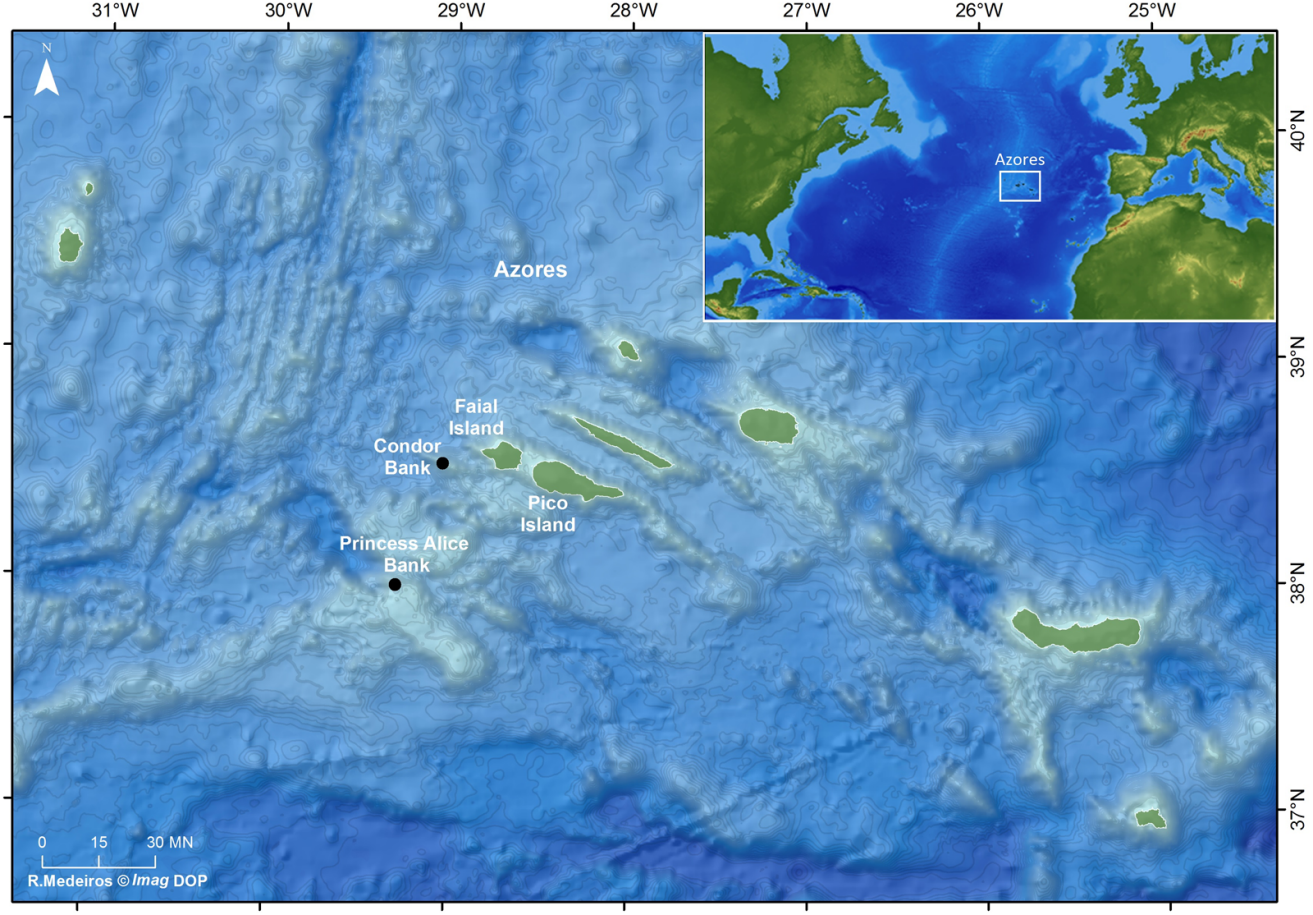
Location of the study sites: Ecological Acoustic Recorders (EARs) deployment locations in the Azores archipelago (black dots) (Ricardo Medeiros @Imag DOP).

The Azorean archipelago is a group of nine volcanic islands located in the North Atlantic Ocean about 1,600 km off the Portuguese continental coast, comprising several seamounts (Fig. 1). The Condor seamount, located about 17 km southwest of Faial Island, is about 1,800 m in height, 39 km long and 23 km wide, extending from a depth of 185 m to 2,003 m. In 2008, Condor was designated as a Scientific Observatory, a protected area for scientific research, through an agreement among local authorities, researchers, fishermen and other stakeholders (Giacomello, Menezes & Bergstad, 2013). Since 2010, demersal fisheries are forbidden, and only the seasonal pole-and-line tuna fishing, big game fishing, eco-touristic (e.g., shark diving) and scientific activities are permitted under a special authorization (Giacomello, Menezes & Bergstad, 2013; Ressurreição & Giacomello, 2013).

Princesa Alice bank is located about 90 km southwest of Pico Island and 80 km southwest of Faial Island (see Fig. 1). The Bank occupies more than 100 km^2^, has a minimum depth of 35 m and maximum depth of 500 m. It is an important fishing area for demersal and pelagic fishes and a popular recreational diving spot.

### Vocal species

A bibliographic research was conducted to identify fish species recorded in the Azores that are either known to be vocal, or that belong to genera and families containing vocal species (Santos, Porteiro & Barreiros, 1997). Several databases and online open access libraries of animal sounds were consulted (e.g., Macaulay Library (2017), DOSITS (2017), The British Library (2017), Fish Base (2017), Chorus Acoustics (2017), see Table S1), to compile a sound database for this region. Sounds were also requested from the authors. However, it should be noted that sounds obtained from different sources may differ in their acoustic parameters due to geographical variations in fish sounds (e.g., Parmentier et al., 2005) and because they may have been recorded in different circumstances such as under different water temperatures; this variability should be considered when comparing sounds recorded in different locations or when building sound databases.

### Acoustic recordings and analysis

Ecological Acoustic Recorders (EARs, Lammers et al., 2008) were bottom-moored on the Condor seamount at an approximate depth of 190 m, 5–10 m from the seafloor, and at a depth of 36 m in Princesa Alice bank, on the seafloor. The EAR is an autonomous acoustic recorder provided with a Sensor Technology SQ26-01 hydrophone that has a flat frequency response (±1.5 dB) from 18 Hz to 28 kHz and a response sensitivity between −193 and −194 dB re 1 V/µPa. (varying between deployments) at Condor and −193.6 dB at Princesa Alice.

From the available deployments, 10 at Condor and 4 at Princesa Alice, we selected recordings from three in Condor (deployments 2, 7 and 10) and from one in Princesa Alice (deployment 3), based on duration and recording quality. The EARs were programmed to record on duty cycles: Condor deployment 2 and 7, 90 s of sound recorded every 900 s at a sampling rate of 50 kHz; deployment 10, 3,600 s every 12,600 s, at 2 kHz; Princesa Alice deployment 3, 90 s every 900 s, at 50 kHz.

Recordings from the following months were analysed. Condor: April, June, August and November 2010; June and August 2012. Princesa Alice: June 2010. From these recordings the Acoustic Complexity Index (ACI) was calculated using the plug-in SounsdscapeMeter in the WaveSurfer software (Pieretti, Farina & Morri, 2011). ACI presents higher values in sounds with inner variability over time (as sounds of biological origin) and low values for more constant sounds (e.g., passing vessels) (Pieretti et al., 2017). Within each month, a subsample of 5 days (24 h periods) was selected from those presenting higher ACI values, expected to be associated with a higher biophony (Pieretti, Farina & Morri, 2011; Bolgan et al., 2018).

**Figure 2:**
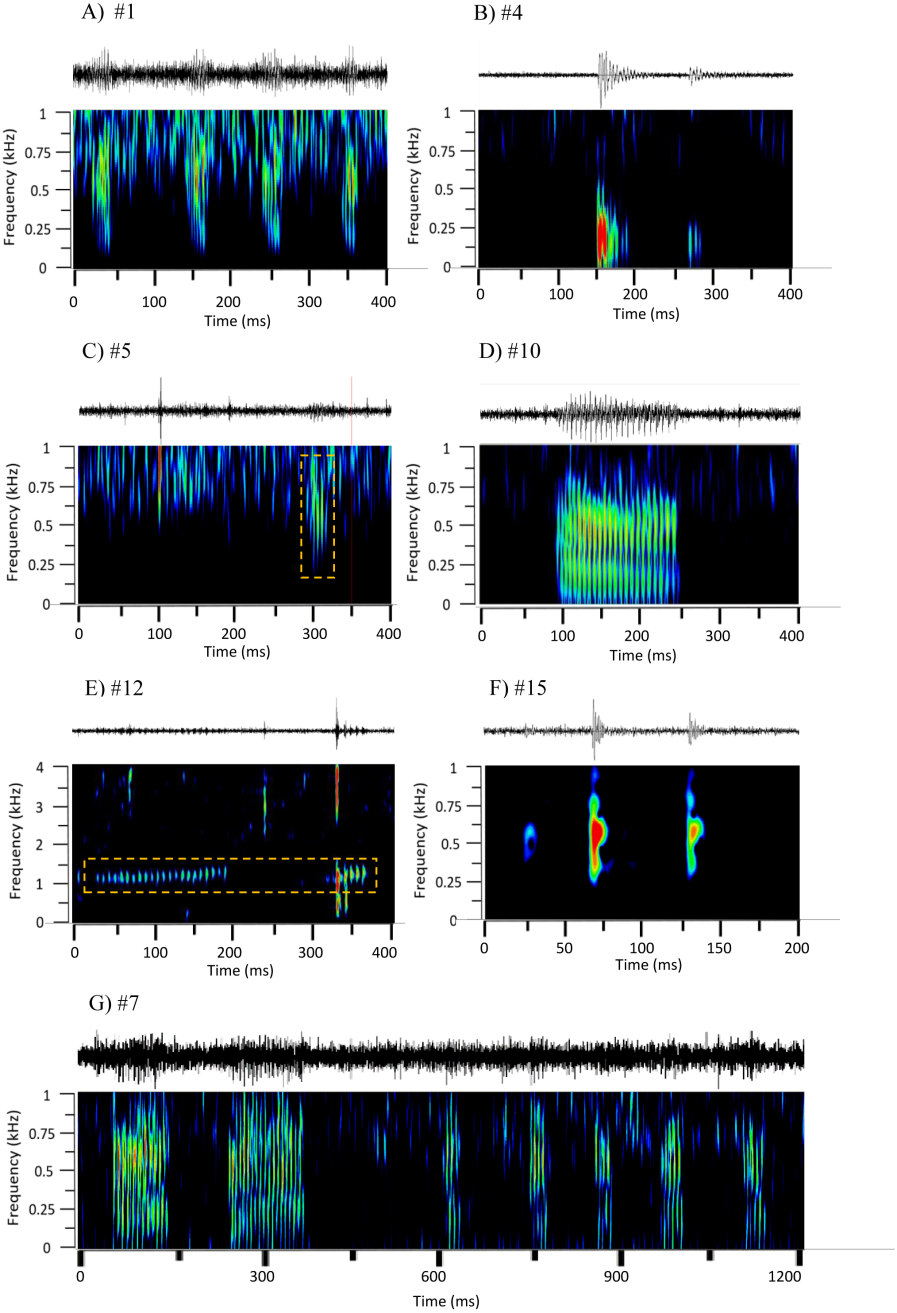
Oscillograms and spectrograms of the seven frequently occurring sound sequences, that were identified as fish calls. Each sound sequence was identified through a # plus number. (A) #1; (B) #4; (C) #5; (D) #10; (E) #12; (F) #15 and (G) #7. Warmer colours indicate higher sound energy. The yellow rectangle helps to highlight the sound in the spectrogram.

**Figure 3:**
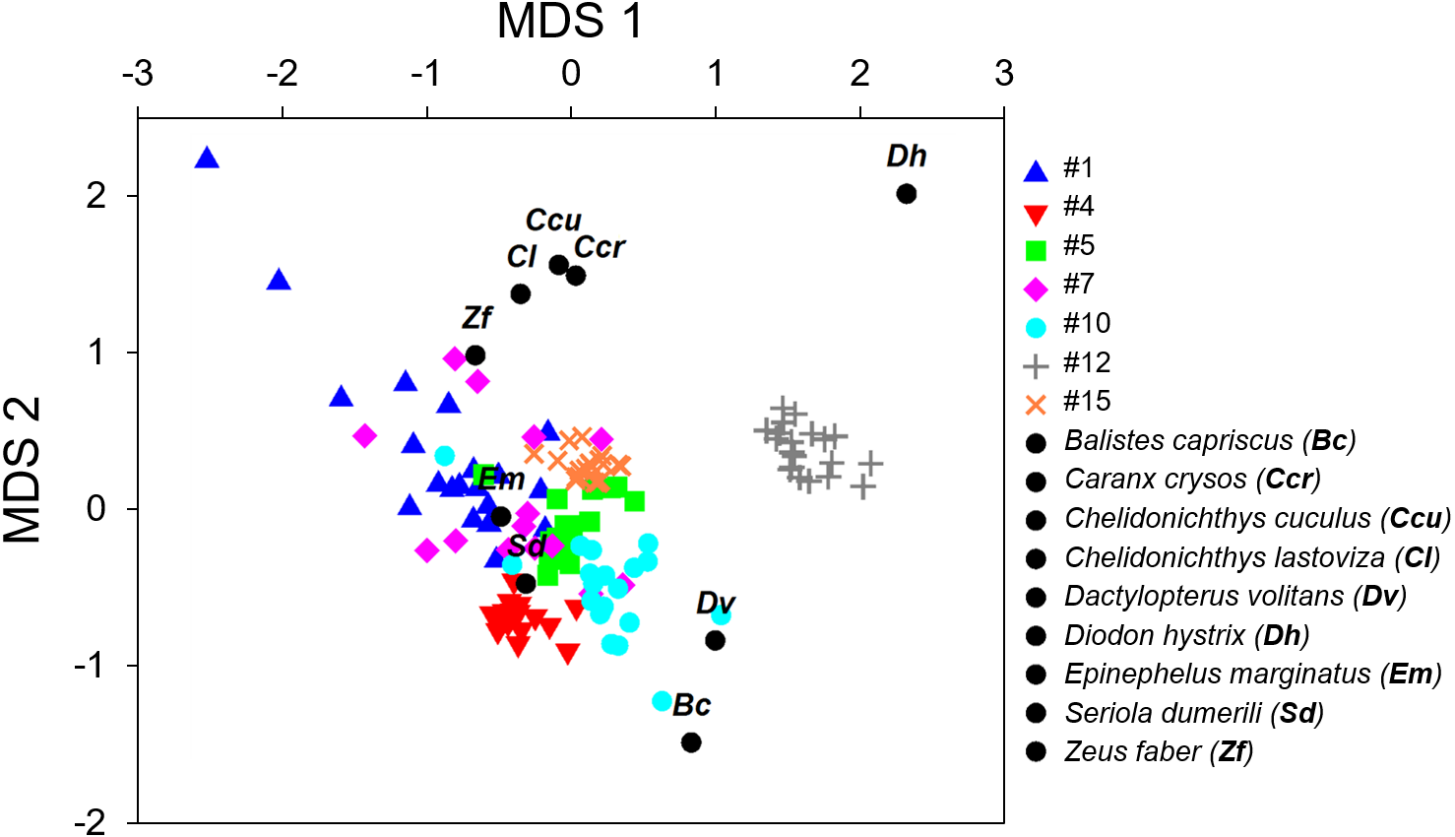
Non-metric multi-dimensional scaling plot (nMDS) of the fish calls exploring the resemblance between the seven recorded sound sequences (Table 4) and the sounds produced by 9 soniferous species that could occur in the surveyed ecosystems.

**Figure 4:**
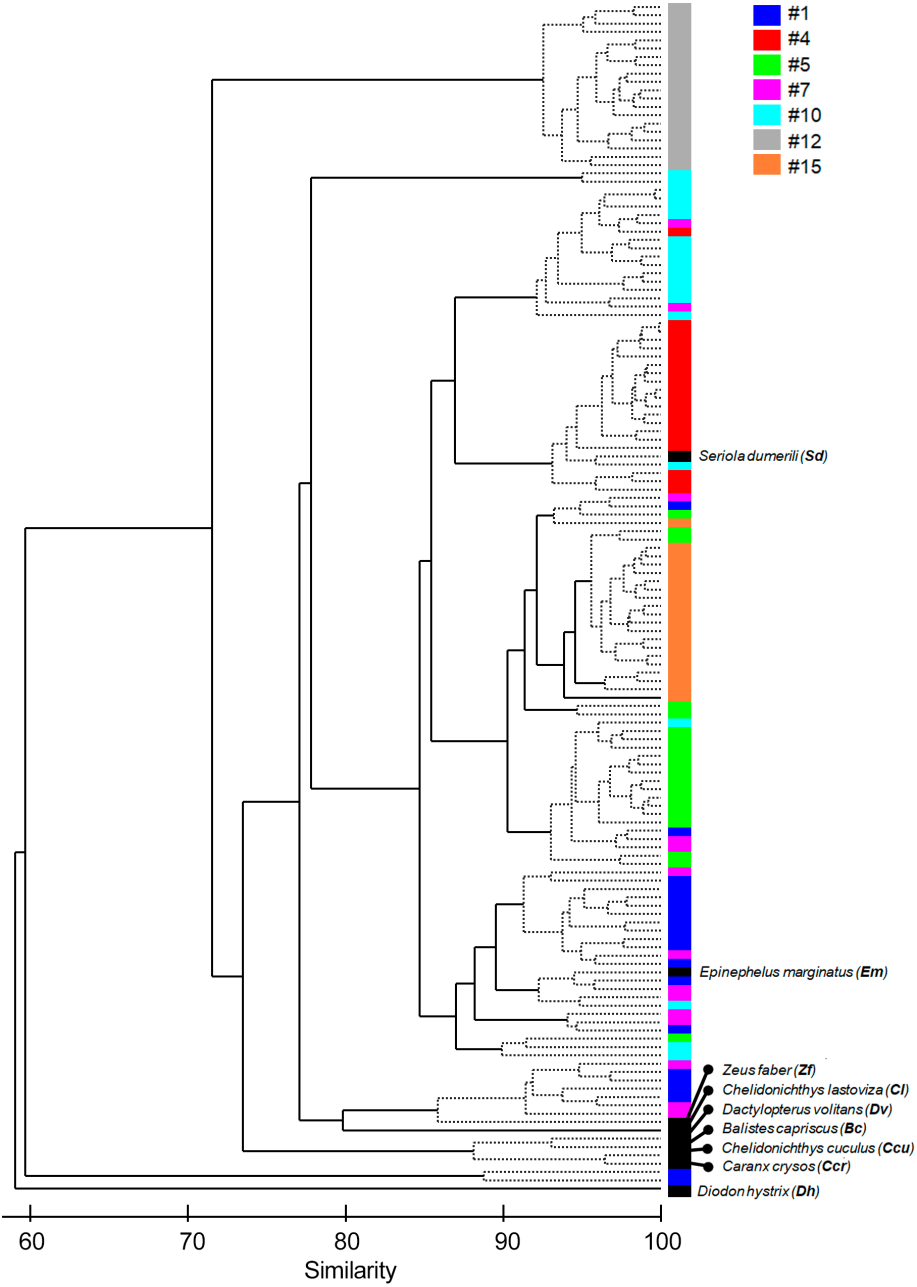
Cluster of the seven most common sound sequences (#1, #4, #5, #7, #10, #12, #15) and sounds from nine identified soniferous species that may occur in the surveyed ecosystems. The Cluster plot shows the degree of similarity between the several sound sequences and the sounds from identified species.

Sound recordings were downsampled to 8 kHz and analyzed with Adobe Audition 3.0, both aurally and visually with spectrograms (FFT 2048 points, Hamming window, frequency range up to 4 kHz, fixed display settings). To inspect low amplitude sounds, a 25 dB amplification was used. Fish sounds were discriminated based on their similarity to reported fish calls, in frequency, relative energy level, duration, and timing (e.g., Parsons et al., 2016a; Parsons et al., 2016b). Because most sounds occurred in sequences and since in many sounds we could not analyse its fine structure due to the likely distance of the sound emitter to the hydrophone, we focussed on identifying sound sequence types rather than sound types. The following parameters were measured for seven types of sound sequences that had at least 14 occurrences, and were identified as fish calls: sequence duration (time elapsed from the start of the first sound in a sequence to the end of the last sound, s); number of sounds; sound duration (the mean duration of a sound in a sequence, s); sound period (mean time elapsed between the peak amplitude of two consecutive sounds within a group, s); peak frequency (frequency at which the sound presents its highest energy in the power spectrum, Hz); minimum and maximum frequency (the lower and the higher frequency of each sound in the spectrogram, Hz) and signal to noise ratio—SNR (ratio between the sound Root Mean Square (RMS) amplitude and the background noise RMS amplitude). Each sound sequence (Fig. 2) was defined based on frequency, duration and temporal patterns. Raven Pro Sound Analysis Software 1.5 (Cornell Lab of Ornithology, USA) was used to measure the temporal acoustic parameters. WaveSurfer was used to draw spectrograms and oscillograms. Fourteen to 20 sound sequence per sound sequence type with SNR > 1.03 were selected and characterized. In addition, 1 sound per soniferous species present in Azores was also characterised and used for comparison with sound sequences. Of the 20 known soniferous species (Table 1) only 9 species were included in this analysis (see Figs. 3 and 4) due to the lack of available sounds. *Pomatoschistus pictus* was not considered since it inhabits shallow waters and its sounds can only be recorded when a fish is very close (a few centimetres) to the hydrophone, thus being unlikely detected by the EARs. Note that caution should be taken when doing this comparison as we only considered one sound per identified vocal species and the analysis is thus not considering intraspecific variability.

We used the software PRIMER 6.0 to explore multivariate similarity profiles among the seven sound sequences and sounds from identified species using a Bray–Curtis Similarity matrix, followed by a Cluster plot and a non-metric multidimensional scaling approach (nMDS) with a 2D Stress of 0.13. The nMDS and the cluster plot were derived from the similarity matrix to evaluate similarities among sound sequences and sounds from known species and to investigate the acoustic parameters that contribute to those similarities (Clarke & Gorley, 2006; Sun et al., 2013). We used a similarity profile test (SIMPROF; Clarke & Warwick, 2001; Clarke, Somerfield & Gorley, 2008) to determine significant differences between the clusters.

The variables used in these multivariate analyses were peak frequency, maximum frequency, minimum frequency, sequence duration, sound duration and number of sounds. Variables were standardized prior to analyses. For this effect the mean was subtracted from each data point and divided by the standard deviation. Two units were further added to eliminate negative values that were not accepted by the analysis.

Since the relation between fish sound abundance and diversity has not been validated for the present study sites, we compared mean ACI values (for the frequencies 15–2,000 Hz) between two months with contrasting fish sound abundance and diversity (August 2009 and June 2010 for Condor).

## Results

### Soniferous fish species present in Azores

We identified 20 soniferous species from the fish occurring in the Azores (Table 1). Other species belonging to the same genera (*n* = 27) or families (*n* = 52) of known vocal species are listed in Table 2. These latter 79 species are potentially sound producers but future studies are needed for confirmation.

The sound sequences produced by the vocal fishes listed in Table 1 are indicated in Table 3 together with the associated behavioural contexts. Examples of these sounds are presented as waveforms and spectrograms in the Supplemental Information (Fig. S1). Available sound files (*n* = 12) are presented in Audio S1 to Audio S12.

### Characterization of fish sounds from Condor and Princesa Alice

In the recordings from Condor seamount and Princesa Alice bank, 20 sound sequences were identified as being likely produced by fishes (Fig. 2, Fig. S2). Seven of these sound sequences (n ≥ 14 occurrences) were characterized quantitatively (Table 4) while the remaining 13 (n <14) were only characterized qualitatively (Table 5, see Supplemental Information for sound files in Audio S13 to Audio S32 and Video S1).

The main characteristics of the different sound sequences detected at Condor seamount and Princesa Alice bank are presented in Tables 4 and 5. Most sounds, were broadband frequency pulse trains with variable spectral range / peak frequency and pulse rates, often produced in sequences. In all of these sounds the pulsed structure could be distinguished by the human ear. From these, sounds #7, #22, #38, #48 stood out. Sound #7 was a sequence of pulse trains with the first elements being more tonal (resembling sound #10); sound #22 had a high spectral range up to 2 kHz; sound #38 presented higher frequency components (1,000–1,400 Hz) and very fast pulse trains with marked amplitude modulation; and #48 showed low frequency components. From the remaining sound sequences, i.e., non-broadband pulse trains, #4, #14, #15 were composed of isolated pulses often in doublets, #10 was more tonal and #12 presented high frequencies and a smaller frequency range.

The multivariate exploratory analyses (Figs. 3 and 4) discriminated sound sequences #4, #5, #10, #12 and #15. These analyses were unable to discriminate between sounds #1, #7 and #10 (Fig. 3). In fact, sound #7 starts with elements that resemble #10 followed by a sequence of sounds that resemble #1. Consistently, the SIMPROF analysis did not reveal significant differences among sound sequences #1, #7 and #10 and showed statistically significant differences among sounds #4, #5, #12 and #15, also supporting our ad-hoc groups of sound sequences that included sounds #1, #5, #7 as broadband pulse trains, #10 as a more tonal sound, #12 as a high frequency sound, and #4 and #15 as isolated pulses.

Comparisons between fish sounds identified in the libraries and the analysed fish sound sequences (Figs. 3 and 4) revealed similarities between the sound emissions of *Dactylopterus volitans* with sound #10, *Seriola dumerilli* with sound #4, as well as of *Epinephelus marginatus* with sounds #1, #5 and #7. However, a more careful inspection of the sounds, made both aurally and by visually analysing the spectrograms and oscillograms, do not support a match with any of the soniferous species available in the inspected databases.

**Figure 5:**
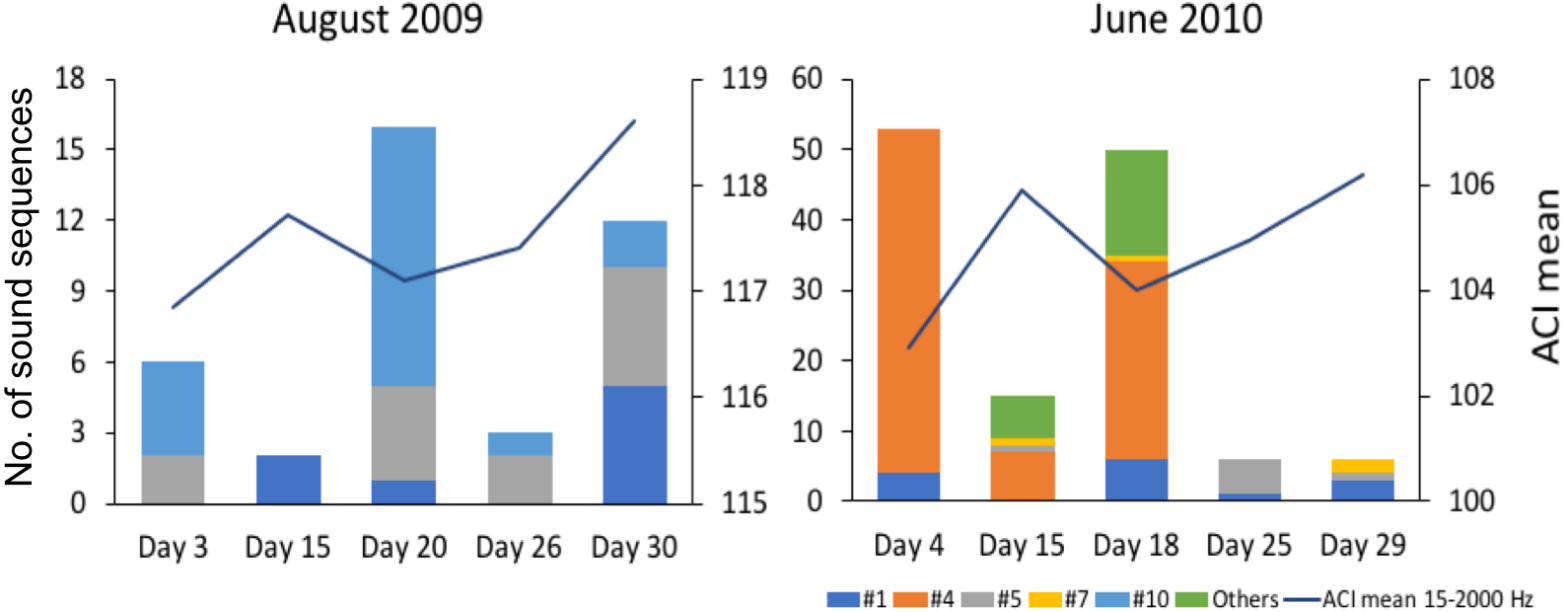
Comparison of mean Acoustic Complexity Index (ACI) values with abundance and diversity of fish sound sequences in two contrasting months. Graphs compare mean Acoustic Complexity Index (ACI) values (line graph) with the abundance and diversity of the seven recorded sequences of sounds (histograms) in August 2009 and June 2010 on the Condor seamount.

The variation in abundance and diversity of sound sequences detected in each of the 5 sampled days in August 2009 and June 2010 in Condor (2 months with contrasting abundance and diversity) did not match the variation in the mean ACI values for the same days (Fig. 5). Inspection of the recordings suggests that ACI values presented higher values when cetacean sounds or boat noise were present within the considered frequency band (15–2,000 Hz). Cetacean sounds and boat noise were discriminated based on aurally and visually analyses of the spectrograms and oscillograms and assessing the respective sound parameters.

## Discussion

Many fish species produce species-specific sounds (Amorim, 2006; Fine & Parmentier, 2015) that can be detected (Vieira et al., 2015) and discriminated (Lillis, Eggleston & Bohnenstiehl, 2014) from other sound sources present in aquatic soundscapes. In this study several fish species present in the Azorean archipelago were identified as sound producers (20 species from 14 families) or potential sound producers (79 species from 24 families) based on the literature. Consistently, we found a considerable diversity of putative fish sounds during the analysis of acoustic recordings from Princesa Alice and Condor, contributing for the marine soundscape of the surveyed Azorean ecosystems. We described 20 sound sequences. From these, seven were sufficiently abundant to be characterized for several acoustic parameters.

**Table 2:** Summary of potentially soniferous species of the Azores.

**Species**^a^	**Family**^a^	**Conservation status**^b^	**Commercial**^c^	**Depth range (m)**^b^^,^^c^	**Similarity**^d^
*Anguilla anguilla* (Eel)	Anguillidae	CR	Commercial; aquaculture; gamefish	0–700	G, F_(2,9)_
*Canthidermis maculatus* (Rough triggerfish)	Balistidae	LC	Commercial	1–110	F_(2,9,10)_
*Blennius ocellaris* (Butterfly blenny)	Blenniidae	LC	Minor commercial	10–100	F_(8,16)_
*Coryphoblennius galerita* (Montagu’s blenny)	Blenniidae	LC	No interest	0–2	F_(8,16)_
*Lipophrys pholis* (Shanny)	Blenniidae	LC	No interest; public aquariums	0–8	F_(8,16)_
*Lipophrys trigloides*	Blenniidae	LC		0–15	F_(8,16)_
*Ophioblennius atlanticus atlanticus* (Redlip blenny)	Blenniidae	LC	No interest; aquarium	0–8	F_(8,16)_
*Parablennius incognitus* (Mystery blenny)	Blenniidae	LC		0–2	G, F_(8,16)_
*Parablennius parvicornis* (Rock-pool blenny)	Blenniidae	LC		0–2	G, F_(8,16)_
*Parablennius ruber* (Portuguese blenny)	Blenniidae	LC		0–20	G, F_(8,16)_
*Seriola rivoliana* (Longfin yellowtail)	Carangidae	LC	Commercial; gamefish	5–245 (30–35)	G, F_(2,9)_
*Decapterus macarellus* (Mackerel scad*)*	Carangidae	LC	Commercial; gamefish; bait	1–400 (40–200)	F_(2,9)_
*Pseudocaranx dentex* (Guelly jack)	Carangidae	LC	Commercial; aquaculture; gamefish	10–238 (10–25)	F_(2,9)_
*Trachurus picturatus* (Blue jack mackerel)	Carangidae	LC	Commercial	305–370	F_(2,9)_
*Sardina pilchardus* (Sardine)	Clupeidae	LC	Highly commercial	10–100	F_(2,9)_
*Gadiculus argenteus argenteus* (Silvery pout)	Gadidade	Not evaluated	Minor commercial; bait: usually	100–1000	F_(3,5,9)_
*Micromesistius poutassou* (Blue whiting)	Gadidade	Not evaluated	Highly commercial	150–3000 (300–400)	F_(3,5,9)_
*Molva macrophthalma* (Spanish ling)	Gadidade	LC	No interest	30–754	F_(3,5,9)_
*Thorogobius ephippiatus* (Leopard-spotted goby)	Gobiidae	LC		6–120	F_(4,7,11)_
*Kyphosus incisor* (Yellow sea chub)	Kyphosidae	Not evaluated	Minor commercial; gamefish	1–15	G, F_(2)_
*Acantholabrus palloni* (Scale-rayed wrasse)	Labridae	LC	Commercial	30–500	F_(9,10,12)_
*Centrolabrus trutta* (Emerald wrasse)	Labridae	LC		1–30	F_(9,10,12)_
*Coris julis* (Rainbow wrasse)	Labridae	LC	Minor commercial; gamefish; aquarium	1–120	F_(9,10,12)_
*Labrus bergylta* (Ballan wrasse)	Labridae	LC	Subsistence fisheries; gamefish; aquarium	1–50	F_(9,10,12)_
*Labrus bimaculatus* (Cuckoo wrasse)	Labridae	LC	Subsistence fisheries; gamefish; public aquariums	20–200 (40–80)	F_(9,10,12)_
*Pseudolepidaplois scrofa* (Barred hogfish)	Labridae	VU	Minor commercial	20–200	F_(9,10,12)_
*Symphodus mediterraneus* (Axillary wrasse*)*	Labridae	LC	Subsistence fisheries; gamefish; aquarium	1–70	F_(9,10,12)_
*Thalassoma pavo* (Ornate wrasse)	Labridae	LC	Minor commercial; gamefish; aquarium	1–150 (1–50)	F_(9,10,12)_
*Xyrichthys novacula* (Cleaver wrasse)	Labridae	LC	Minor commercial; gamefish; aquarium	1–90	F_(9,10,12)_
*Masturus lanceolatus* (Sharptail mola)	Molidae	LC		0–670	F_(2)_
*Ranzania laevis* (Slender sunfish)	Molidae	LC		1–140	F_(2)_
*Mullus surmuletus* (Red mullet)	Mullidae	LC	Commercial; gamefish	5–409	F_(2)_
*Manta birostris* (Giant manta)	Myliobatidae	VU	Minor commercial	0–1000	F_(2)_
*Mobula mobular* (Devil ray)	Myliobatidae	EN		0–700	F_(2)_
*Myliobatis aquila* (Eagle ray)	Myliobatidae	DD	Minor commercial; gamefish	1–300	F_(2)_
*Brotulotaenia brevicauda*	Ophidiidae	Not evaluated	No interest	0–2650	F_(1,9,14)_
*Brotulotaenia crassa* (Violet cuskeel)	Ophidiidae	Not evaluated	No interest	249–1100	F_(1,9,14)_
*Holcomycteronus squamosus*	Ophidiidae	LC	No interest	1147–5055	F_(1,9,14)_
*Monomitopus metriostoma*	Ophidiidae	LC	No interest	235–1570	F_(1,9,14)_
*Parophidion vassali*	Ophidiidae	DD	No interest		F_(1,9,14)_
*Spectrunculus grandis* (Pudgy cuskeel)	Ophidiidae	LC	Minor commercial	800–4300	F_(1,9,14)_
*Acanthostracion notacanthus* (Island cowfish)	Ostraciidae	DD		3–25 (?-10)	F_(2,10)_
*Gaidropsarus granti* (Azores rockling)	Phycidae	DD	Commercial	120–823	G, F_(3)_
*Gaidropsarus guttatus* (Spotted rockling)	Phycidae	DD	Subsistence fisheries	0–20	G, F_(3)_
*Gaidropsarus mauli* (Deep sea rockling)	Phycidae	Not evaluated		900–1700	G, F_(3)_
*Phycis blennoides* (Greater forkbeard)	Phycidae	Not evaluated	Commercial	10–1308	F_(3)_
*Phycis phycis* (Forkbeard)	Phycidae	LC	Minor commercial	13–614	F_(3)_
*Chromis limbata* (Azores chromis)	Pomacentridae	LC	Minor commercial	5–45	G, F_(7,9,12)_
*Sparisoma cretense* (Parrotfish)	Scaridae	LC	Commercial	2–50	G, F_(10,12)_
*Helicolenus dactylopterus* (Blackbelly rosefish)	Scorpaenidae	LC	Commercial	20–1100	F_(2,9)_
*Pontinus kuhlii* (Offshore rockfish)	Scorpaenidae	DD	Commercial	91–600	F_(2,9)_
*Scorpaena azorica*	Scorpaenidae	Not evaluated			G, F_(2,9)_
*Scorpaena laevis* (Senegalese rockfish)	Scorpaenidae	LC	Commercial	1–100	G, F_(2,9)_
*Scorpaena maderensis* (Madeira rockfish)	Scorpaenidae	LC	Commercial	20–40	G, F_(2,9)_
*Scorpaena notata* (Small red scorpionfish)	Scorpaenidae	LC	Commercial; aquarium	10–700	G, F_(2,9)_
*Scorpaena porcus* (Black scorpionfish)	Scorpaenidae	LC	Minor commercial; aquarium	10–90	G, F _(2,9)_
*Scorpaena scrofa* (Red scorpionfish)	Scorpaenidae	LC	Commercial; public aquariums	20–200	G, F_(2,9)_
*Setarches guentheri* (Channeled rockfish)	Scorpaenidae	LC	Minor commercial	150–800	F_(2,9)_
*Trachyscorpia cristulata echinata* (Spiny scorpionfish)	Scorpaenidae	DD	Commercial	140–2230	F_(2,9)_
*Anthias anthias* (Swallowtail seaperch)	Serranidae	LC	Commercial; gamefish; aquarium	15–300	F_(9,12,13)_
*Mycteroperca fusca* (Island grouper)	Serranidae	EN	Commercial	1–200 (20–30)	G, F_(9,12,13)_
*Serranus atricauda* (Blacktail comber)	Serranidae	DD	Commercial	2–90	G, F_(9,12,13)_
*Serranus cabrilla* (Comber)	Serranidae	LC	Minor commercial; gamefish; aquarium	1–450	G, F_(9,12,13)_
*Boops boops* (Bogue)	Sparidae	LC	Highly commercial; gamefish; bait usually	0–350	F_(2,9)_
*Diplodus sargus cadenati* (Moroccan white seabream)	Sparidae	LC	Commercial	1–150	G, F_(2,9)_
*Pagellus acarne* (Axillary seabream)	Sparidae	LC	Commercial; gamefish	40–500 (40–100)	F_(2,9)_
*Pagellus bogaraveo* (Blackspot seabream)	Sparidae	NT	Commercial; gamefish: yes	1–800	F_(2,9)_
*Pagrus pagrus* (Red porgy)	Sparidae	LC	Commercial; aquaculture; gamefish; aquarium	0–250 (10–80)	F_(2,9)_
*Sarpa salpa* (Salema)	Sparidae	LC	Commercial; gamefish; bait occasionally	5–70	F_(2,9)_
*Sphyraena viridensis* (Yellowmouth barracuda)	Sphyraenidae	LC	Commercial	0–100	G, F_(2)_
*Entelurus aequoreus* (Snake pipefish)	Syngnathidae	LC	No interest; public aquariums	5–100	F_(2)_
*Hippocampus hippocampus* (Short-snouted seahorse)	Syngnathidae	DD	Minor commercial; public aquariums	?–60	G, F_(2)_
*Hippocampus ramulosus* (Seahorse)	Syngnathidae	DD	Minor commercial; aquarium	1–20	G, F_(2)_
*Nerophis maculatus* (Spotted Pipefish)	Syngnathidae	DD		?–30	F_(2)_
*Syngnathus acus* (Greater pipefish)	Syngnathidae	LC	No interest	0–110 (3–12)	F_(2)_
*Lagocephalus lagocephalus* (Oceanic puffer)	Tetraodontidae	LC	Commercial; gamefish	10–476 (10–100)	G, F_(2,9)_
*Sphoeroides marmoratus* (Guinean puffer)	Tetraodontidae	LC	Commercial	1–100	G, F_(2,9)_
*Sphoeroides pachygaster* (Blunthead puffer)	Tetraodontidae	LC	Commercial	50–480 (50–250)	G, F_(2,9)_
*Zenopsis conchifer* (Sailfin dory)	Zeidae	LC	Commercial	50–600 (150–300)	F_(6,9,15)_

**Notes.**

a Marine fishes of the Azores—Annotated checklist and bibliography.

b IUCN Red List of Threatened Species (2017)

c http://www.fishbase.org/

d Sound production references: 1, Courtenay (1971); 2, Fish & Mowbray (1970); 3, Almada et al. (1996); 4, Zelick, Mann & Popper (1999); 5, Hawkins & Amorim (2000); 6, Onuki & Somiya (2004); 7, Amorim (2006); 8, De Jong, Bouton & Slabbekoorn (2007); 9, Kasumyan (2008); 10, Parmentier et al. (2011); 11, Parmentier et al. (2013); 12, Tricas & Boyle (2014); 13, Bertucci et al. (2015); 14, Parmentier & Fine (2016); 15, Radford, Putland & Mesinger (2018); 16, Ladich (2019).

Conservation status from IUCN: LC, Least Concern; VU, Vulnerable; NT, Near Threatened; EN, Endangered; CR, Critically Endangered; DD, Data deficient. Depth range with most frequent depths presented in brackets. Commercial status: indication of the degree of commercial interest referring to fisheries followed by other types of commercialization; denoted when available. Similarity: G, fish species belonging to the same genus of known sound-producing species; F, fish species belonging to the same family of known sound-producing species.

Exploratory multivariate analyses based on the Bray-Curtis Similarity index agree with an ad-hoc classification made through comparison of the sound spectrograms and by listening to the sounds, suggesting this method produced a valid classification. The only exception being sound #7 that presented similarities with sounds #1 and #10 regarding their spectrograms (Figs. 3 and 4). This suggests that these three sound sequences might belong to closely related species, the same species or even be variants of the same sound type. The remaining sounds are more likely to be produced by different species. In particular sound #12, with energy at higher frequencies, appears to be quite different from the other groups. We cannot ensure, however, that all the identified sound sequences were made by fish, although they exhibited general characteristics common in fish sounds, i.e., they were low frequency short duration sounds, with no frequency modulation, and temporal patterns within the range of other reported sounds (e.g., Fish & Mowbray, 1970; Amorim, 2006; Parsons et al., 2016a; Parsons et al., 2016b).

**Table 3:** Sound production in Azorean fishes and their behavioral context. Same species from Table 1.

**Species**	**Sound type**	**Behavioural context**	**References**
*Abudefduf luridus* (Canary damsel)	Single-pulse, two pulses, train of pulses	Agonistic	Santiago & Castro (1997)
*Chelidonichthys cuculus* (Red gurnard)	Knocks, grunts and growls	Agonistic	Amorim (1996)
*Balistes capriscus* (Grey triggerfish)	Toothy grunts; low thumps	During manual and electric stimulation	Fish & Mowbray (1970)
*Canthigaster rostrata* (Sharpnose puffer)	n/a	Moderate sound under manual stimulation	Fish & Mowbray (1970)
*Caranx crysos* (Blue runner)	Weak scrapes, loud grunts	Under duress	Fish & Mowbray (1970)
*Dactylopterus volitans* (Flying gurnard)	n/a	Strong sound under manual stimulation	Fish & Mowbray (1970)
*Diodon hystrix* (Spot-fin porcupinefish)	Defensive inflation with associated sounds of jaw stridulation	Feeding	Fish & Mowbray (1970)
*Elagatis bipinnulata* (Rainbow runner)	n/a	Under manual stimulation	Fish & Mowbray (1970)
*Epinephelus marginatus* (Dusky grouper)	Single booms, serial booms, growls	Courtship	Bertucci et al. (2015)
*Gobius paganellus (* Rock goby*)*	Tonal sounds	Agonistic, courtship	Malavasi, Collatuzzo & Torricelli (2008)
*Kyphosus sectatrix* (Bermuda sea chub)	Grunts, thumps, knocks	Alarm calls	Fish & Mowbray (1970)
*Mola mola* (Ocean sunfish)	n/a	Strong sound under manual stimulation	Fish & Mowbray (1970)
*Naucrates ductor* (Pilotfish)	n/a	n/a	Fish (1954)
*Pomatomus saltatrix* (Blue fish)	Escape sounds, clicks & thumps	Under duress	Fish & Mowbray (1970)
*Pomatoschistus pictus* (Painted goby)	Thump and drums	Agonistic, courtship	Amorim & Neves (2008)
*Scorpaena plumieri* (Spotted scorpionfish)	n/a	Weak sound under stimulation	Fish & Mowbray (1970)
*Seriola dumerili* (Greater amberjack)	Thumps & knocks	Competitive feeding	Fish & Mowbray (1970)
*Trachinotus glaucus* (Pompano)	n/a	Weak sound under manual and electric stimulation	Fish & Mowbray (1970)
*Chelidonichthys lastoviza (* Streaked gurnard*)*	Knocks and growls	Competitive feeding	Amorim (1996)
*Zeus faber (* John dory*)*	Low frequency growl, barks	Agonistic	Onuki & Somiya (2004)

**Notes.**

n/a not available

Previous studies have shown that the Acoustic Complexity Index (ACI) is a useful tool to track variations in the soundscapes (e.g., Pieretti et al., 2017). Our results showed that higher values of the ACI might not indicate a higher abundance and diversity of fish sounds since we did not observe a relation between the abundance and diversity of fish sounds and the ACI values (Fig. 5). In fact, the ACI apparently increased in the presence of cetacean sounds or boat noise. Although this index is known to respond well to biological sounds in recordings with low background noise, it may fail in the presence of anthropogenic or environmental noise (Lin, Fang & Tsao, 2017), under continuous noise such as that created by fish choruses (Bolgan et al., 2018), or in the case reported here where the fish sounds are sparse and with a low signal to noise ratio. In fact, better results can be obtained when using ACI in combination with other methods (Phillips, Towsey & Roe, 2018). This paper supports the approach that multiple acoustic indices are required to understand a soundscape.

**Table 4:** Average (±Standard deviation (SD) and range) values of measured acoustic variables for the seven types of sound sequences that had at least 14 occurrences, and were identified as fish calls. A spectrogram is presented in Fig. 2 for each group.

**Sound sequence ID**	**Recording site**	**N**	**Peak frequency (Hz)**	**Min frequency (Hz)**	**Max frequency (Hz)**	**Sequence Duration (s)**	**Sound duration (s)**	**SoundPeriod (s)**	**N° of sounds**
#1	Condor; P. Alice	20	318.8 ± 131.3 (125–625)	94.2 ± 25.1 (55–148)	662.6 ± 162.1 (430–1109)	3.45 ± 2.97 (0.62–13.14)	0.05 ± 0.02 (0.03–0.09)	0.27 ± 0.09 (0.18–0.48)	16 ± 17 (4-72)
#4	Condor; P. Alice	20	131.3 ± 28.0 (125–250)	57.8 ± 18.5 (31–94)	300.2 ± 87.8 (172–539)	0.23 ± 0.12 (0.06–0.41)	0.09 ± 0.04 (0.04–0.19)	0.22 ± 0.03 (0.15–0.27)	2 ± 0.5 (1-2)
#5	Condor; P. Alice	20	462.5 ± 146.8 (250–875)	149.2 ± 68.4 (86–313)	736.6 ± 158.9 (492–1008)	0.39 ± 0.84 (0.03–3.81)	0.06 ± 0.02 (0.03–0.10)	0.47 ± 0.42 (0.20–1.23)	2 ± 0.8 (1-4)
#7	Condor; P. Alice	14	357.1 ± 201.3 (125–750)	74.4 ± 20.2 (47–117)	713.3 ± 215.2 (367–1094)	2.12 ± 1.61 (0.49–5.64)	0.10 ± 0.06 (0.03–0.24)	0.31 ± 0.11 (0.18–0.56)	8 ± 6.6 (2-23)
#10	Condor; P. Alice	20	350 ± 104.2 (250–625)	89.2 ± 20.8 (55–133)	630.5 ± 190.9 (391–1031)	0.78 ± 1.20 (0.12–5.09)	0.22 ± 0.09 (0.06–0.46)	0.75 ± 0.43 (0.35–1.37)	2 ± 1.1 (1-5)
#12	Condor; P. Alice	20	1175 ± 85.1 (1000–1375)	919.9 ± 100.3 (805–1094)	1468.8 ± 139.6 (1266–1727)	0.56 ± 0.26 (0.09–1.01)	0.21 ± 0.07 (0.09–0.38)	0.44 ± 0.10 (0.30–0.68)	2 ± 0.7 (1-3)
#15	P. Alice	20	543.8 ± 73.4 (375–625)	238.0 ± 40.4 (141–297)	922.7 ± 118.0 (750–1219)	0.52 ± 0.38 (0.11–1.62)	0.02 ± 0.004 (0.02–0.03)	0.16 ± 0.05 (0.09–0.27)	4 ± 1.5 (2-7)

**Table 5:** Description of the 20 new sound sequences characterized.

Sound sequence	ID Recording site	Description
#1	Condor; P. Alice	Series of relatively short (<50 ms) trains of pulses with broadband frequency and peak frequency of about 300 Hz
#2	Condor; P. Alice	Tonal sound with a frequency range of 20–1,200 Hz. Fundamental frequency of about 100 Hz. Peak frequency at about 200 Hz and a duration of 51 ms
#3	Condor; P. Alice	Isolated pulse train and tonal with frequency range of 300–600 Hz, fundamental frequency at about 350 Hz and peak frequency of about 500 Hz. Duration of about 600 ms
#4	Condor; P. Alice	Broadband sound with two pulsed portions and a peak frequency about 100 Hz
#5	Condor; P. Alice	Similar to sound #1, one or two isolated broadband pulse-trains with a peak frequency of about 450 Hz
#6	Condor	Tonal sound with a frequency range of 100–200 Hz, a fundamental frequency at about 150 Hz, and a duration of about 400 ms
#7	Condor; P. Alice	Long pulse trains followed by shorter trains, similar to sound #1. Peak frequency of about 350 Hz
#8	Condor	Long pulsed sound followed by 4 or 5 shorter pulsed elements. Broadband with a frequency range of 300–800 Hz, main frequency about 350 Hz. and a duration about 600 ms
#10	Condor; P. Alice	Mostly a tonal sound, with a fundamental frequency at about 100 Hz and a peak frequency at about 350 Hz
#12	P. Alice	Two similar elements. Peak frequency at about 1,100 Hz
#14	P. Alice	Group of double short elements; pulsed sound, including one or two pulses, frequency range of 100–1,200 Hz, with a peak frequency of 350–450 Hz and a duration of about 400 ms
#15	Condor; P. Alice	Pulsed sound. Set of 4 pulses grouped two by two. Peak frequency of about 550 Hz
#17	P. Alice	Broadband pulse train composed by 11 or 12 pulses, with a frequency range of 20–900 Hz. Duration of about 200 ms
#22	P. Alice	Broadband sound, with a frequency range of 20–2,000 Hz. Duration about 200 ms
#28	P. Alice	Series of 4 pulse trains, broadband consisting each of a sequence of 4 pulses. Frequency range of 50–300 Hz. Duration of 1.5 s
#35	P. Alice	Pulse train and a broadband sound, with a frequency range of 20–1,400 Hz. Peak frequency of 400–500 Hz and a duration of about 200 ms
#38	P. Alice	High frequency pulsed broadband sound, with a frequency range of 700–1,700 Hz. Peak frequency of 1,000–1,400 Hz and a duration of about 1.3
#47	P. Alice	Broadband pulsed sound composed by groups of two pulses and a frequency range of 100–1,400 Hz. It lasts about 500 ms
#48	P. Alice	Broadband pulse train with 1 or 2 pulses. Frequency range of 50–600 Hz. It has a duration of about 350 ms
#50	Condor	Broadband sound with a peak frequency of 350 Hz and with a duration of about 1.1 s

All 20 sound-producing fish species present in the Azores partially or fully overlap their depth distribution range with the study sites. Of the 79 species occurring in the Azores that potentially produce sounds only six are unlikely to be found in either study sites due to their depth distribution range, all others thus being potential source candidates for the recorded fish sounds. Known acoustic detection distances are usually short for fish, so detected sounds can be assumed to have been produced relatively close to the recorders. Indeed, fish sound propagation distances can vary from a few centimeters (e.g., gobies; Lugli & Fine, 2003) to a few meters (e.g., toadfish and damselfish; Fine & Lenhardt, 1983; Myrberg Jr, Mohler & Catala, 1986; Alves, Amorim & Fonseca, 2016; sweeper; Radford et al., 2015). Exceptions are sciaenids which have been estimated to be detectable from tens to few hundred meters (Locascio & Mann, 2011; Parsons et al., 2012). Most of the above acoustic detection distances, however, have been reported for fish calling in shallow water, thus facing strong propagation constraints due to the frequency cutoff phenomenon (Rogers & Cox, 1988). In deeper waters such as Condor, fish sounds will likely propagate to longer distances than in shallow water (Mann, 2006) but proximity to the recorder will still be a constraint for monitoring most soniferous species.

Can any of the described sound sequences be attributed to a known vocal fish species? Comparisons made between the reference fish sounds and the seven recorded sound sequences (Figs. 3 and 4) must be considered with care since only one sound from each species was available. We found some resemblance in the analysis between some sound sequences (e.g., #4 with *Seriola dumerilli,* sounds #1, #5 and #7 with *Epinephelus marginatus,* the sound #10 with *Dactylopterus volitans*) but a more careful inspection revealed that none of these species sounds matched the identified sound sequences. Sound sequences #1, #5, #7 and #10 displayed similarities with sounds produced by species of Batrachoididae, which generate tonal sounds with several harmonics and dominate soundscapes in different habitats across the world (Bass & McKibben, 2003; Maruska & Mensinger, 2009; Rice et al., 2016). Sound sequences #1, #5 are similar to grunt trains, #10 resembles boatwhistles from toadfishes and #7 resembles boatwhistles followed by grunts trains. For example, advertisement calls of *Opsanus beta* are composed by grunt and tonal elements (Thorson & Fine, 2002). No Batrachoididade species are known for the Azores, though *Halobatrachus didactylus* occurs in the eastern Atlantic, and both *Opsanus tau* and *Opsanus phobetron* in the western Atlantic (Amorim, 2006; Amorim, Simões & Fonseca, 2008; Fine, 1978). Sound sequence #1 sounds like an Ophidiiform (M Bolgan, pers. comm., 2019). Sound sequence #12 (duration: 0.56 s, peak frequency: 1,175 Hz; Table 4) exhibits similarities with the /kwa/ recorded in *Posidonia oceanica* meadows in the Mediterranean Sea, recently attributed to *Scorpaena* spp. (0.27s duration and 747 Hz peak frequency; Di Iorio et al., 2018; Bolgan et al., 2019). This sound presents several acoustic features which are typical of the “kwa” sound. In particular, peak frequency is always higher than 600 Hz, the pulse envelop presents a highly stereotyped amplitude modulation where cycle period corresponds to peak frequency; finally, the sound presents a typical pseudo-harmonic structure where the pseudo-harmonic interval corresponds to the inverse of the pulse period (Di Iorio et al., 2018; Bolgan et al., 2019). Interestingly sound sequence #12 also shows some resemblance in frequency and duration with the chatter sound made by cusk-eels *Ophidion marginatum* (Ophidiidae) (Sprague & Luczkovich, 2001; Mann & Jarvis, 2004), although they do not seem similar to the human ear. Both members of the Scorpaenidae and Ophidiidae can be found in Azores (Table 2).

Although we could not suggest a potential candidate for sound sequence #15, similar sounds were also found during boat-based recordings around Faial Island at depths between 2–10 m (R Carriço, pers. comm., 2017) indicating that its emitter may also inhabit shallow waters. Sound sequences #1, #2, #3, #4, #5, #7, #10 and #15 were recorded in both study locations, suggesting a wide depth distribution. In contrast, sounds #6 and #8 were recorded only in Condor (190 m) while sounds #12, #14, #17, #22, #28, #35, #38, #47 and #48 were recorded only in Princesa Alice (36 m) indicating that these soniferous fishes may be somewhat stenobathic.

Potential vocal fish species present in Condor at the studied depth (Table 2) produce sounds similar to the sound sequences that were recorded. For example, *Phycis phycis*, *Phycis blennoides*, *Anthias anthias*, and *Molva macrophtalma* are potential source species for the calls #6 and #8, while *Pagellus bogaraveo* and *Helicolenus dactylopterus* are potential source species for the sound sequences #1, #2, #3, #4, #5, #7, #10 or #15 (Menezes & Giacomello, 2013). On the other hand, *Sphyraena viridensis* and *Seriola rivoliana* have confirmed presence in Princesa Alice (Fontes & Afonso, 2017), being eventually potential source species for the sound sequences #12, #14, #17, #22, #28, #35, #38, #47 or #48.

## Conclusions

The present study highlights the wealth of fish sounds awaiting to be described and challenges associated with PAM. For example, only some soniferous fish species produce loud conspicuous sounds easily detectable by PAM. Also most fish sounds are still unidentified making it difficult to identify the sources of most fish sounds detected with PAM. However, these monitoring studies can contribute to evaluate fish presence and abundance, to identify spawning seasons of species of commercial, conservation and scientific interest. It can also be used to investigate fish community structure (Harris, Shears & Radford, 2015; McWilliam & Hawkins, 2013). To increase the effectiveness of PAM, basic research is needed on sound source identity, behavioral context of sounds production, and spatial and temporal distribution of the sounds/species (Rountree et al., 2006; Sirovic et al., 2009; Wall et al., 2014). Two important approaches to overcome these main challenges are coupling PAM with *in situ* visual monitoring techniques (e.g., Mouy et al., 2018), and recording more species in laboratory conditions.

Our results provide baseline data on a collection of sounds, contributing to building a comprehensive open access library of both identified and unknown fish sounds that will boost the usefulness of PAM.

##  Supplemental Information

10.7717/peerj.7772/supp-1 Supplemental Information 1 Video with the sound files and spectrograms of the twenty fish sound sequences found in Azores

10.7717/peerj.7772/supp-2 Supplemental Information 2 Sound file of Red gurnard - Chelidonichthys cuculus

10.7717/peerj.7772/supp-3 Supplemental Information 3 Sound file of Streaked gurnard - Chelidonichthys lastoviza

10.7717/peerj.7772/supp-4 Supplemental Information 4 Sound file of Grey triggerfish - Balistes capriscus

10.7717/peerj.7772/supp-5 Supplemental Information 5 Sound file of Blue runner - Caranx crysos

10.7717/peerj.7772/supp-6 Supplemental Information 6 Sound file of Flying gurnard - Dactylopterus volitans

10.7717/peerj.7772/supp-7 Supplemental Information 7 Sound file of Porcupine fish - Diodon hystrix

10.7717/peerj.7772/supp-8 Supplemental Information 8 Sound file of Dusky grouper - Epinephelus marginatus

10.7717/peerj.7772/supp-9 Supplemental Information 9 Sound file of Blue fish - Pomatomus saltatrix

10.7717/peerj.7772/supp-10 Supplemental Information 10 Sound file of Greater amberjack - Seriola dumerili

10.7717/peerj.7772/supp-11 Supplemental Information 11 Sound file of Painted goby - Pomatoschistus pictus (thump)

10.7717/peerj.7772/supp-12 Supplemental Information 12 Sound file of Painted goby - Pomatoschistus pictus (drum)

10.7717/peerj.7772/supp-13 Supplemental Information 13 Sound file of fish sound sequence #1

10.7717/peerj.7772/supp-14 Supplemental Information 14 Sound file of fish sound sequence #2

10.7717/peerj.7772/supp-15 Supplemental Information 15 Sound file of fish sound sequence #3

10.7717/peerj.7772/supp-16 Supplemental Information 16 Sound file of fish sound sequence #4

10.7717/peerj.7772/supp-17 Supplemental Information 17 Sound file of fish sound sequence #5

10.7717/peerj.7772/supp-18 Supplemental Information 18 Sound file of fish sound sequence #6

10.7717/peerj.7772/supp-19 Supplemental Information 19 Sound file of fish sound sequence #7

10.7717/peerj.7772/supp-20 Supplemental Information 20 Sound file of fish sound sequence #8

10.7717/peerj.7772/supp-21 Supplemental Information 21 Sound file of fish sound sequence #10

10.7717/peerj.7772/supp-22 Supplemental Information 22 Sound file of fish sound sequence #12

10.7717/peerj.7772/supp-23 Supplemental Information 23 Sound file of fish sound sequence #14

10.7717/peerj.7772/supp-24 Supplemental Information 24 Sound file of fish sound sequence #15

10.7717/peerj.7772/supp-25 Supplemental Information 25 Sound file of fish sound sequence #17

10.7717/peerj.7772/supp-26 Supplemental Information 26 Sound file of fish sound sequence #22

10.7717/peerj.7772/supp-27 Supplemental Information 27 Sound file of fish sound sequence #28

10.7717/peerj.7772/supp-28 Supplemental Information 28 Sound file of fish sound sequence #35

10.7717/peerj.7772/supp-29 Supplemental Information 29 Sound file of fish sound sequence #38

10.7717/peerj.7772/supp-30 Supplemental Information 30 Sound file of fish sound sequence #47

10.7717/peerj.7772/supp-31 Supplemental Information 31 Sound file of fish sound sequence #48

10.7717/peerj.7772/supp-32 Supplemental Information 32 Sound file of fish sound sequence #50

10.7717/peerj.7772/supp-33 Supplemental Information 33 Fish sounds databases List of databases and online open access libraries of animal sounds including fish.

10.7717/peerj.7772/supp-34 Supplemental Information 34 Oscillograms and spectrograms of vocal fish species present in the Azorean archipelago Oscillograms and spectrograms of vocal fish species present in the Azorean archipelago: (A) Red gurnard (Amorim, 1996); (B) Streaked gurnard (Amorim, 1996); (C) Grey triggerfish (Macaulay Library); (D) Blue runner (Fish Base); (E) Painted goby –drum (Amorim & Neves, 2008); (F) Painted goby –courtship thump (Amorim & Neves, 2008); (G) John dory (Mensinger et al., 2016); (H) Flying gurnard (Macaulay Library); (I) Porcupine fish (Macaulay Library); (J) Dusky grouper (Bertucci et al., 2015); (K) Blue fish (Fish Base) and (L) Greater amberjack (Fish Base). Spectrograms were created using a 2048 points FFT with a Hamming window from wav files. Warmer colours indicate higher sound energy

10.7717/peerj.7772/supp-35 Supplemental Information 35 Oscillogram and spectrogram of other less abundant identified fish calls detected in the Azorean archipelago Oscillogram and spectrogram of other less abundant identified fish calls detected in the Azorean archipelago, Portugal: A –#2; B - #3; C - #6; D - #8; E - #14; F - #17; G - #22; H - #28; I - #35; J - #38; K - #47; L - #48 and M - #50. Spectrograms were created using a 2,048 points FFT with a Hamming window from wav files recorded at 50 kHz. Warmer colours indicate higher sound energy.

## Additional Information and Declarations

### Data Availability

The following information was supplied regarding data availability: The sound files and video are provided in the Supplemental Information: Audio S1–S32; Video S1. Audio S1–S11 includes eleven sounds from the ten vocal species available and Audio S13–S32 includes the twenty fish sound sequences recorded in Azores. Video S1 provides the spectrograms and sounds from the twenty fish sound sequences recorded in Azores.
