# Supplementary figures and images for: Characterization of the acoustic community of vocal fishes in the Azores
Source: PeerJ. 2019 Nov 4;7:e7772. doi: 10.7717/peerj.7772 (PMC6836754; doi:10.7717/peerj.7772)

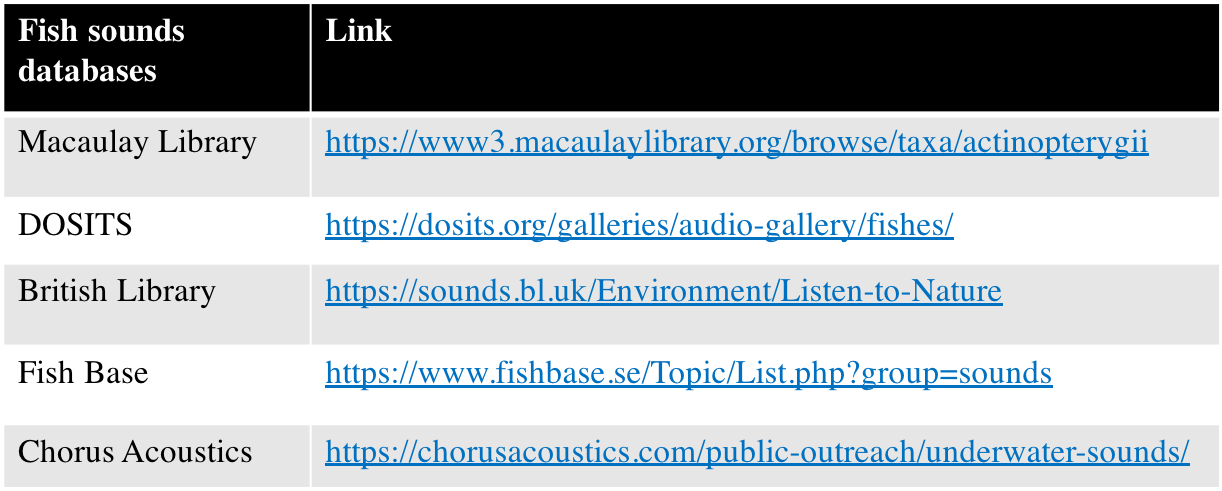

Supplement: Supplemental Information 33 — List of databases and online open access libraries of animal sounds including fish. [file peerj-07-7772-s033.docx]

A) 2#

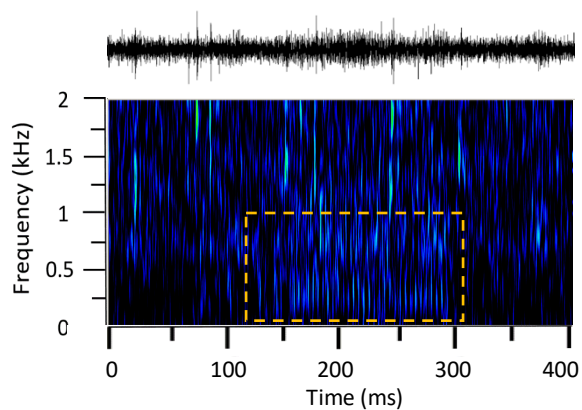

B) #3

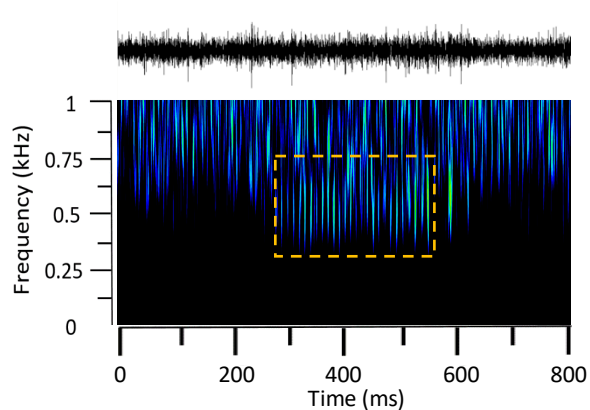

C) #6

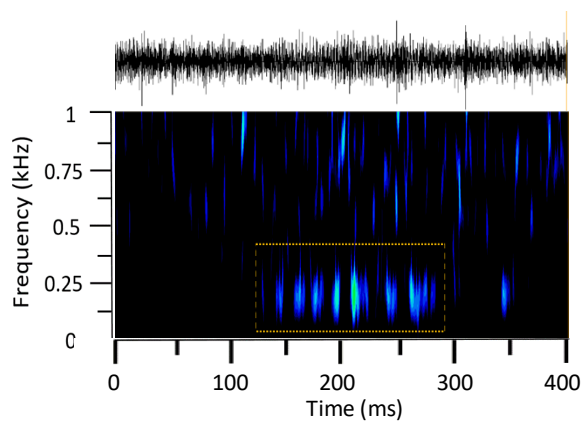

D) #8

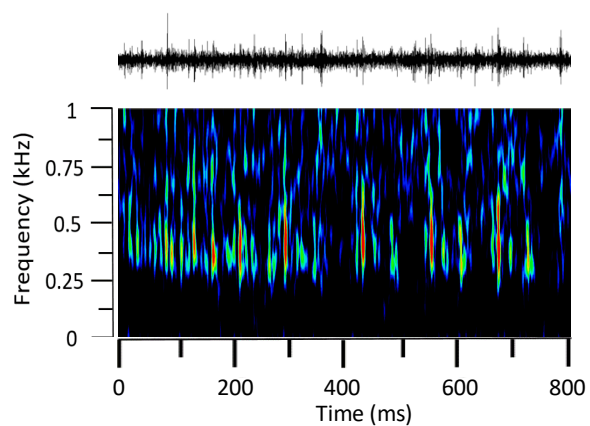

E) #14

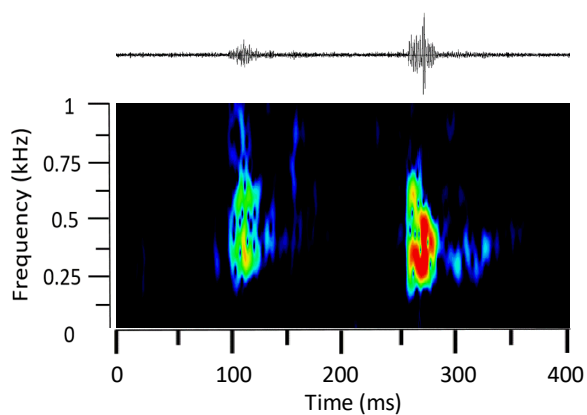

F) #17

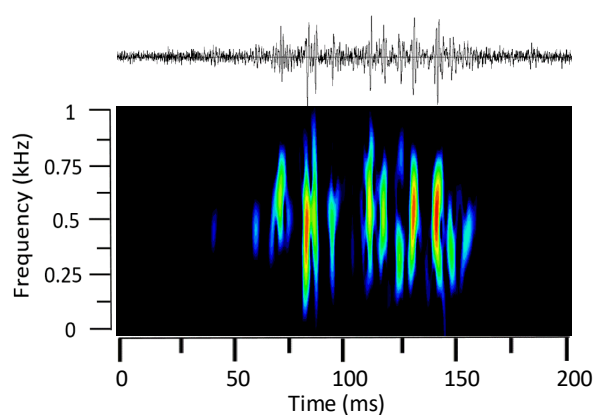

G) #22

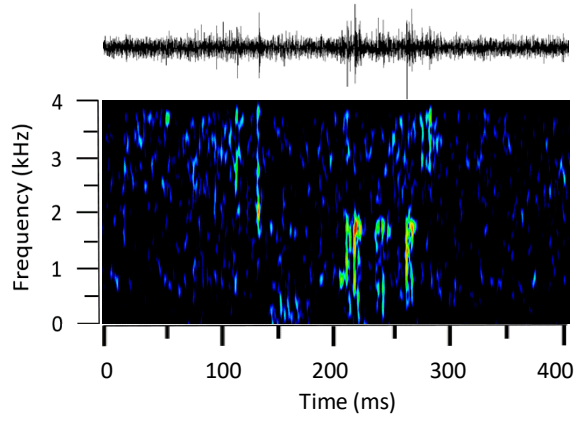

H) #28

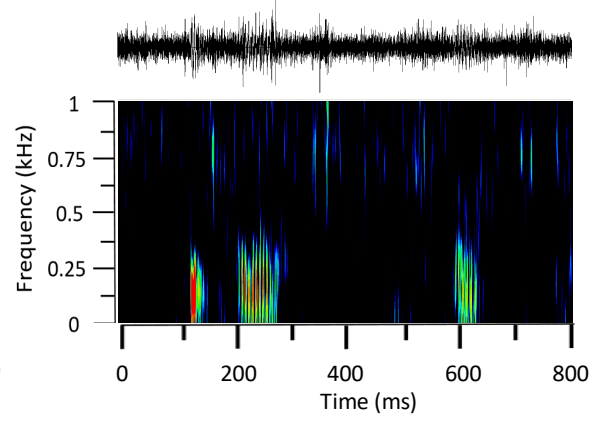

I) #35

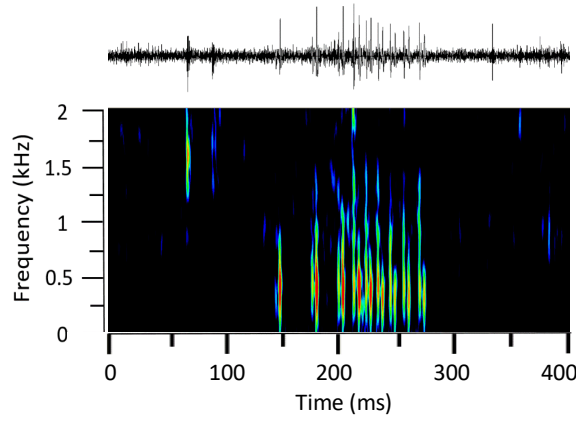

J) #38

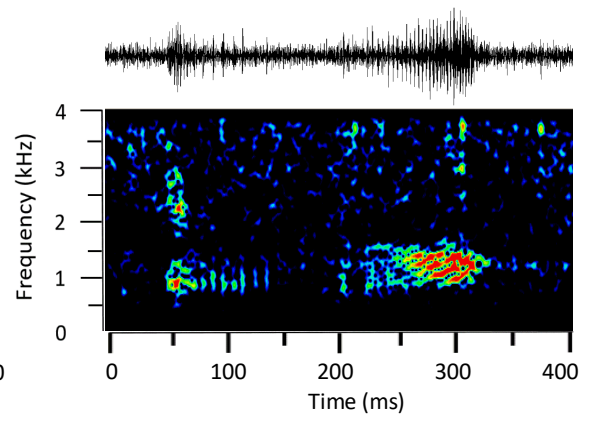

K) #47

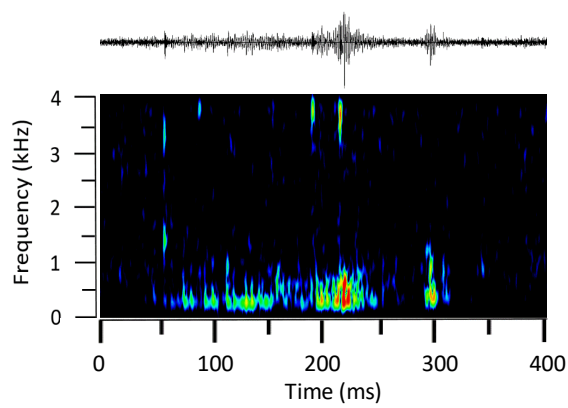

L) #48

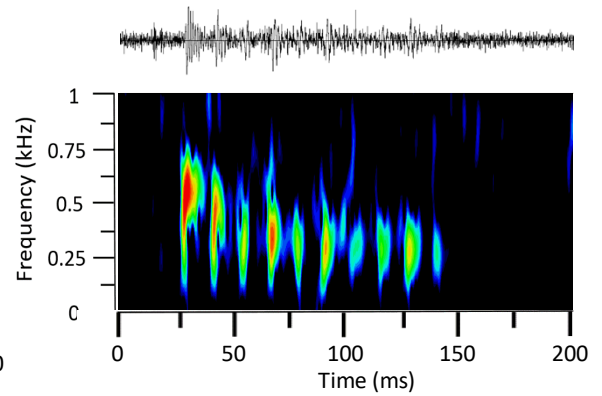

M) #50

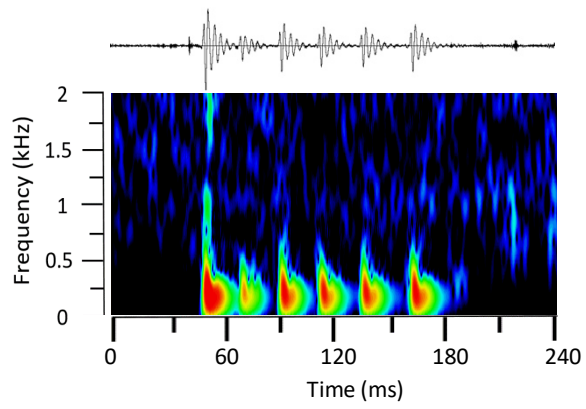

Supplement: Supplemental Information 35 — Oscillogram and spectrogram of other less abundant identified fish calls detected in the Azorean archipelago, Portugal: A –#2; B - #3; C - #6; D - #8; E - #14; F - #17; G - #22; H - #28; I - #35; J - #38; K - #47; L - #48 and M - #50. Spectrograms were created using a 2,048 points FFT with a Hamming window from wav files recorded at 50 kHz. Warmer colours indicate higher sound energy. [file peerj-07-7772-s035.pdf]
