# Supplementary material for: Characterization of the acoustic community of vocal fishes in the Azores
Source: PeerJ. 2019 Nov 4;7:e7772. doi: 10.7717/peerj.7772 (PMC6836754; doi:10.7717/peerj.7772)

A) Red gurnard - *Chelidonichthys cuculus*

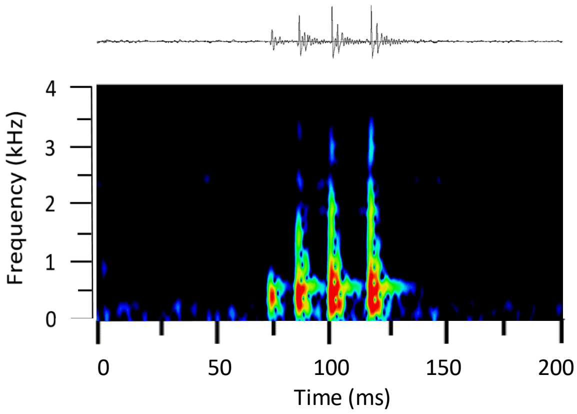

B) Streaked gurnard - *Chelidonichthys lastoviza*

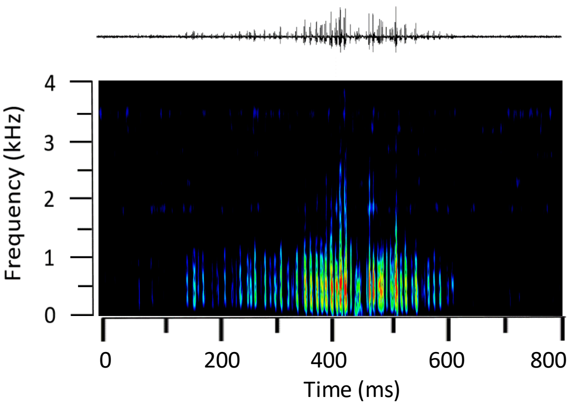

C) Grey triggerfish – *Balistes capriscus*

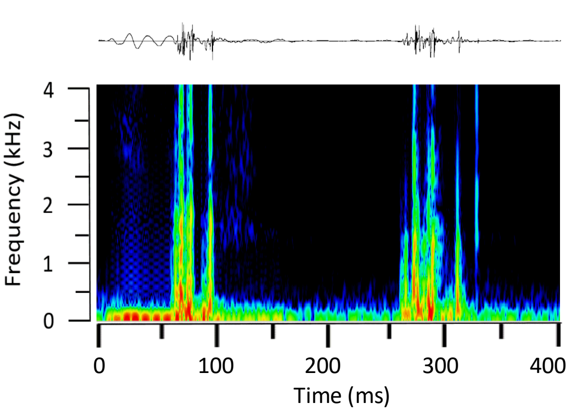

D) Blue runner – *Caranx crysos*

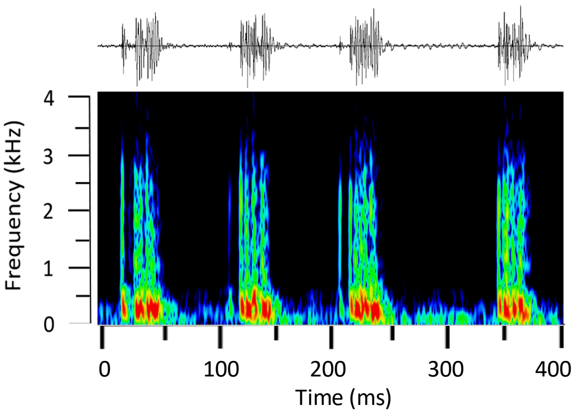

E) Painted goby – *Pomatoschisctus pictus* (drum)

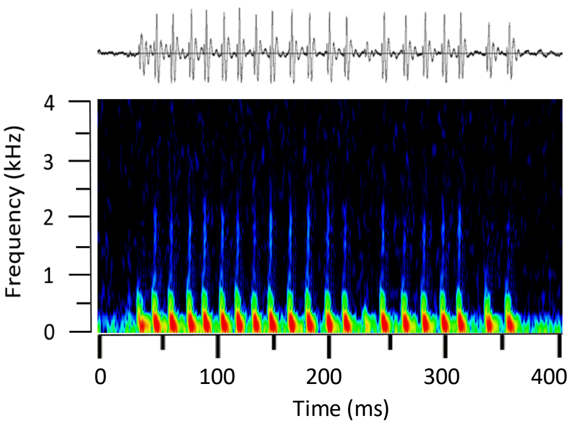

F) Painted goby –*Pomatoschisctus pictus* (thump)

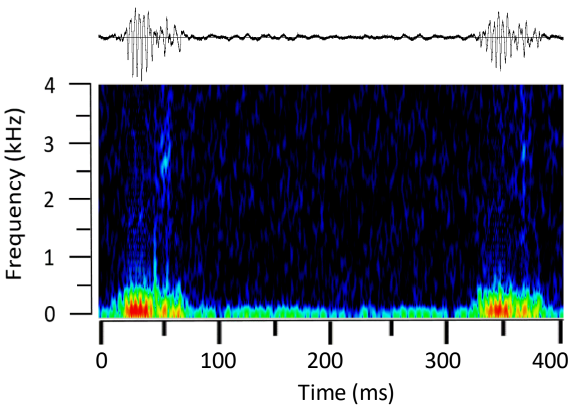

G) John dory - *Zeus faber*

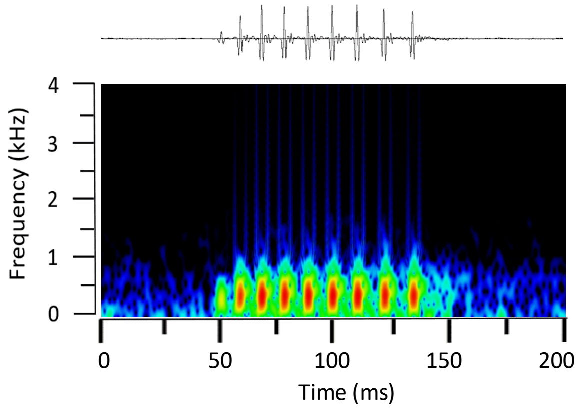

H) Flying gurnard - *Dactylopterus volitans*

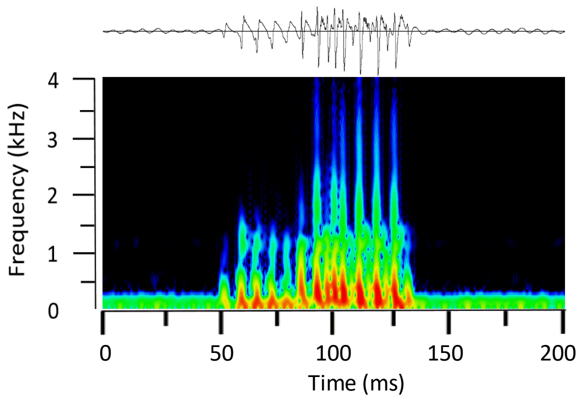

I) Porcupine fish - *Diodon hystrix*

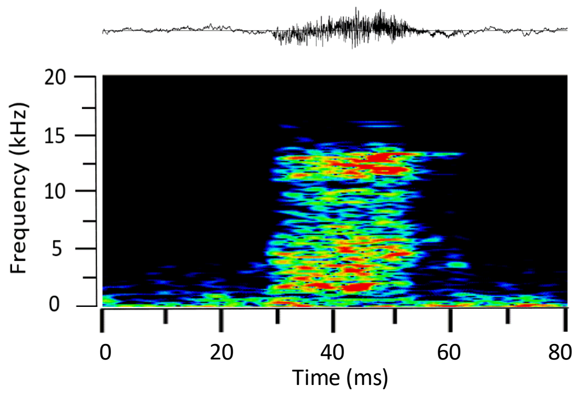

J) Dusky grouper - *Epinephelus marginatus*

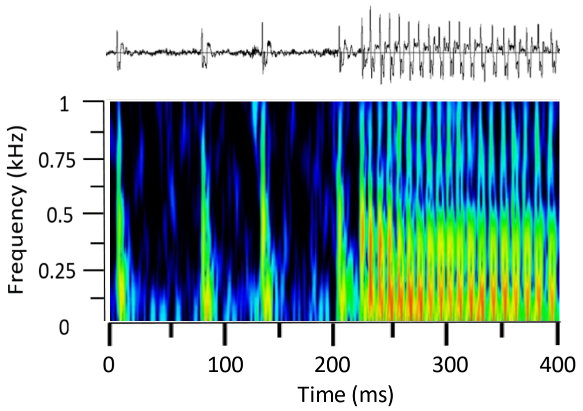

K) Blue fish - *Pomatomus saltatrix*

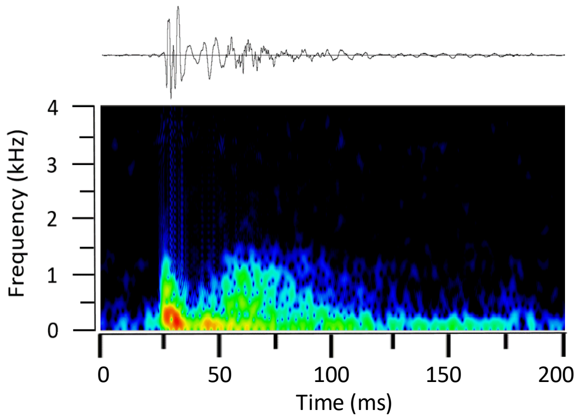

L) Greater amberjack - *Seriola dumerili*

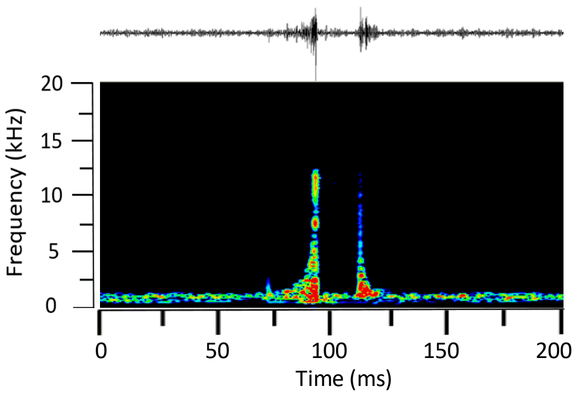

Supplement: Supplemental Information 34 — Oscillograms and spectrograms of vocal fish species present in the Azorean archipelago: (A) Red gurnard (Amorim, 1996); (B) Streaked gurnard (Amorim, 1996); (C) Grey triggerfish (Macaulay Library); (D) Blue runner (Fish Base); (E) Painted goby –drum (Amorim & Neves, 2008); (F) Painted goby –courtship thump (Amorim & Neves, 2008); (G) John dory (Mensinger et al., 2016); (H) Flying gurnard (Macaulay Library); (I) Porcupine fish (Macaulay Library); (J) Dusky grouper (Bertucci et al., 2015); (K) Blue fish (Fish Base) and (L) Greater amberjack (Fish Base). Spectrograms were created using a 2048 points FFT with a Hamming window from wav files. Warmer colours indicate higher sound energy [file peerj-07-7772-s034.pdf]
